# The Story of the Insane from Year to Year

**Published:** 1899-12-09

**Authors:** 


### THE STORY OF THE INSANE FROM YEAR TO YEAR.

Twelfth Series.

### PLYMOUTH BOROUGH ASYLUM.

From this (the seventh annual) report we learn that the asylum contained 249 patients on January 1st, 1898, and that on the last day of the year the number had risen to 269. There were 105 first admissions, 3 readmissions, 34 recoveries, and 23 deaths. The recoveries stand at 33·66 per cent. on the admissions, and the deaths at 8·67 per cent. on the average number resident. Of the deaths, 39 were caused by cerebral and spinal diseases, 14 by consumption, 4 by bronchitis, 3 by pneumonia, and 1 by pleurisy. This gives a total of 22 deaths from lung affections in an asylum containing under 300 patients, and in addition we notice that there were 5 deaths from tuberculosis. It would be very desirable to have an explanation of this high mortality. Of the admissions, 10 owed their insanity to various moral causes, 9 to intemperance in drink, 16 to hereditary tendency, and 20 to previous attacks. It was not considered necessary to use mechanical restraint during the year, but seclusion was used in the cases of 12 men and 8 women; and we are pleased to see that Dr. Bowes does not neglect this useful adjunct to other methods of treatment. The medical superintendency changed hands since the sixth report was printed. Dr. Davis was promoted to the Devon County Asylum at Exminster. He had been superintendent of the Borough Asylum since its opening, and his able administration there has been more than once acknowledged by the committee of visitors. Dr. Bowes, who had been for seven years assistant medical officer, was unanimously appointed to fill the vacancy, and the visitors say that "he has shown himself to be a conscientious, careful, economical, and able administrator." The weekly cost of maintenance was 11s. 1⅝d. per head.

### HEREFORD COUNTY ASYLUM AT BURGHILL.

Here the numbers were almost stationary, being 360 on January 1st and 363 on December 31st. There were 65 first admissions, 16 readmissions, 25 recoveries, 8 discharged relieved, and 28 deaths. The recoveries stand at 32 per cent. of the admissions, and the deaths at 7·5 of the average number resident. There were 12 deaths from cerebral and spinal diseases, 1 from pneumonia, and 1 from consumption. Of the year's admissions 15 were driven insane by adverse circumstances, domestic trouble, and religion, 1 by intemperance in drink, 17 by hereditary influence, and 10 by previous attacks. It is a fact that the public, and even the medical profession at large, are extremely ignorant of the routine of our county asylums; of the amount of medical work which is quietly prosecuted in them; and of the endless sources of anxiety ever present. Dr. Morrison refers to this in a paragraph of his report, from which we extract the following: "It must have occurred to every medical officer of an asylum how lamentably our Asylum Tables fail to show the relation of things; as for instance, in exhibiting the ages at admission and of those resident, the figures but inadequately describe the amount of care, sick nursing, and personal attention the helpless and feeble old folk demand, and the anxiety they occasion." And Dr. Morrison might say similar things of every one of his statistical tables. The visitors are spending £22,500 on enlarging the asylum. It is strange these enlargements do not include a small detached hospital for infectious diseases; and, as Dr. Morrison puts it, the want of an infectious hospital will leave the equipment of this asylum deficient when larger numbers come into residence. The clerk and storekeeper retired owing to ill-health, and received a pension of £130 a year. Very wisely, the two posts will now be held by two officers. The committee "express their satisfaction with the successful management of the asylum by the medical superintendent." The weekly cost of maintenance is 8s. 10½d. per head.

### THE DERBY COUNTY ASYLUM AT MICKLEOVER.

During last year the numbers dropped from 595 to 591. There were 138 first admissions, 25 re-admissions, 63 recoveries, and 84 deaths. The recoveries are 40·1 per cent. of the admissions, and the deaths 13·9 per cent. of the average number resident. Of the year's deaths, 33 were due to cerebral and spinal affections, 22 to consumption, 4 to pneumonia, 3 to bronchitis, and 7 to colitis. Of the year's admissions, 21 owed their insanity to such moral causes as domestic trouble, adverse circumstances, and mental anxiety, 15 to intemperance in drink, 33 to hereditary influence, and 25 to previous attacks. As already stated, the death rate is high, considerably higher than the County Asylum average, and also higher than the Derby average; and it will be noticed that consumption and dysentery figure largely in the death returns. Regarding the consumption, we are inclined to hazard a guess that the outdoor exercise of the patients is insufficient, and, above all, that they are not supplied with 3,000 cubic feet of fresh air per hour each during the night. It has been demonstrated over and over again that the most delicate patients may inhale air of almost any degree of coldness if only sufficient bed clothing be supplied; but unfortunately it is only too true that patients and even nurses will close windows and ventilators at night unless these are placed beyond the control of patients and nurses. Otherwise it is highly probable that the minimum of 3,000 cubic feet of fresh air per hour is not supplied and disease appears sooner or later. As regards colitis it has broken out in more than half the asylums in England, and most medical superintendents can echo Dr. Legge's words that "no light has been thrown upon the origin of the epidemic." Dr. Barwise, medical officer of health, made an exhaustive examination of the sanitary arrangements and failed to detect any fault sufficient to account for the outbreak. Dr. Barwise's report is a very able one, and we earnestly commend it to all medical superintendents, more especially that paragraph on page 20 in which he points out that the organism which causes the colitis may take root on polluted soil or befouled wood, and may lie dormant for any length of time until suitable climatic conditions occur for its development. This is almost certainly true as regards the recurrence and propagation of this troublesome disease, but does not account for its first appearance. In all asylums, too, it has been noticed that patients are attacked in an altogether higher relative proportion than the nurses. At Derby it would seem that 32 patients were attacked and not a single nurse. We are glad to note that the nursing lectures have been continued during the year and that 19 of the staff obtained the medico-psychological certificate, but we do not hear how many, if any, failed to obtain it. We congratulate attendant William Hill on having received his pension. The maintenance rate is a fraction under 10s. per head per week.

